# Real-time Controlling Dynamics Sensing in Air Traffic System

**DOI:** 10.3390/s19030679

**Published:** 2019-02-07

**Authors:** Yi Lin, Xianlong Tan, Bo Yang, Kai Yang, Jianwei Zhang, Jing Yu

**Affiliations:** 1 National Key Laboratory of Fundamental Science on Synthetic Vision, Sichuan University, Chengdu 610065, China; scu_lyi@stu.scu.edu.cn (Y.L.); yangkai@scu.edu.cn (K.Y.); yu_j@scu.edu.cn (J.Y.); 2 National Key Laboratory of Air Traffic Control Automation System Technology, Sichuan University, Chengdu 610065, China; 3 Southwest Air Traffic Management Bureau, Civil Aviation Administration of China, Chengdu 610000, China; caactxl@sina.com

**Keywords:** ATC speech, automatic speech recognition, controlling instruction understanding, deep learning, language model, average pooling

## Abstract

In order to obtain real-time controlling dynamics in air traffic system, a framework is proposed to introduce and process air traffic control (ATC) speech via radiotelephony communication. An automatic speech recognition (ASR) and controlling instruction understanding (CIU)-based pipeline is designed to convert the ATC speech into ATC related elements, i.e., controlling intent and parameters. A correction procedure is also proposed to improve the reliability of the information obtained by the proposed framework. In the ASR model, acoustic model (AM), pronunciation model (PM), and phoneme- and word-based language model (LM) are proposed to unify multilingual ASR into one model. In this work, based on their tasks, the AM and PM are defined as speech recognition and machine translation problems respectively. Two-dimensional convolution and average-pooling layers are designed to solve special challenges of ASR in ATC. An encoder–decoder architecture-based neural network is proposed to translate phoneme labels into word labels, which achieves the purpose of ASR. In the CIU model, a recurrent neural network-based joint model is proposed to detect the controlling intent and label the controlling parameters, in which the two tasks are solved in one network to enhance the performance with each other based on ATC communication rules. The ATC speech is now converted into ATC related elements by the proposed ASR and CIU model. To further improve the accuracy of the sensing framework, a correction procedure is proposed to revise minor mistakes in ASR decoding results based on the flight information, such as flight plan, ADS-B. The proposed models are trained using real operating data and applied to a civil aviation airport in China to evaluate their performance. Experimental results show that the proposed framework can obtain real-time controlling dynamics with high performance, only 4% word-error rate. Meanwhile, the decoding efficiency can also meet the requirement of real-time applications, i.e., an average 0.147 real time factor. With the proposed framework and obtained traffic dynamics, current ATC applications can be accomplished with higher accuracy. In addition, the proposed ASR pipeline has high reusability, which allows us to apply it to other controlling scenes and languages with minor changes.

## 1. Introduction

Air traffic is a complex time-varying, and highly human-dependent system, in which ground-based air traffic controllers (ATCOs) provide required services to guide the aircraft to its destination safely [1]. The primary requirement of air traffic system is to prevent collisions, organize and expedite the air traffic flow, and provide required information and other support for pilots. Surveillance infrastructures, i.e., radar, ADS-B, etc., are built to collect real-time air traffic situation which is displayed in air traffic control systems (ATCSs). ATCOs monitor the air traffic operation and direct aircraft crew to avoid potential conflicts by changing its flight profile, which further ensures the safety and order of the air traffic. ATCOs send the controlling instruction to corresponding pilot by a VHF (very high frequency) voice communication system [2]. That is to say, the communication speech between ATCOs and pilots (air traffic control (ATC) speech) contains the real-time controlling intent and its basic parameters, i.e., controlling dynamics, which implies the trend of air traffic evolution in the near future. Unfortunately, the ATC speech has not been fully utilized in current systems and is only stored for the post incident analysis. With introducing the ATC speech, ATCSs can monitor the ATC process to reduce human errors and obtain real-time traffic information to support ATC decision-making in advance, such as the trajectory prediction, conflict detection and resolution, and flow management [3]. Therefore, it is urgent to develop efficient techniques to process the ATC speech to obtain real-time controlling dynamics. To achieve this goal, the automatic speech recognition (ASR) is the first step and the core bridge between ATCOs and ATCSs.

Automatic speech recognition has been studied for many years. Speech in the time domain is usually converted to frequency the domain to extract diverse acoustic features, such as Mel-frequency cepstral coefficients (MFCCs) [4]. The most classic ASR model is the Hidden Markov Model and Gaussian Mixture Model (HMM/GMM)-based ones [5]. HMM is applied to build the state transition of the sub-phonetic sequence, while GMM is used to predict measurement probabilities of HMM states given the input feature of the speech frame. About 20 years ago, deep neural network (DNN) was introduced to ASR system to replace the GMM, i.e., HMM/DNN model [6], which obtained higher recognition accuracy compared to HMM/GMM models due to the excellent non-linearity modeling of DNN on acoustic features. Although the performance of HMM/DNN-based models is impressive, it still has a complicated pipeline and is a highly expert-dependent work. Recently, thanks to the proposed Connectionist Temporal Classification (CTC) loss function [7], the end-to-end ASR system came into being and achieved compelling superior performance over HMM-based models [8]. The mapping from variable length speech frames to variable length labels can be realized by CTC automatically, which eliminates the complicated process pipeline of traditional methods. Many state-of-the-art ASR models were proposed based on the long-short term memory (LSTM) and CTC architecture, such as CLDNN [9], deep convolutional neural networks (CNN) [10], Deep Speech [11], and Network-in-Network [12].

In the early stage of our research, we attempted to use some existing ASR models to translate the ATC speech. However, all models failed to solve this issue with an acceptable performance due to following specificities of the ATC speech, which provide many challenges to translate them into texts in practice.

(a) Complex background noise: the ATC speech is transmitted by radiophone system which causes some unexpected noises and the ambient noise of ATCOs’ office also affects the intelligibility of the speech [13]. Different application scenes make the noise more diverse and complicated, which is hard to be filtered. Based on the open THCHS30 (Mandarin Chinese) [14] and TED-lium (English) [15] corpus, we use the PESQ [16] to evaluate the quality of the training samples used in this work. Based on a reference speech (THCHS30 and TED-lium in this work), the PESQ gives a score between 1 (poor) and 4.5 (good) for evaluating the quality of speech, and 3.8 is the acceptable score for the telephone voice. Finally, the measurements for the Chinese and English speech used in this work are 3.359 and 3.441 respectively.
(b) High speech rate: the speech rate in ATC is higher than that of speech in daily life since ATC requires high timeliness.
(c) Code switching: to eliminate the misunderstanding of ATC speech, the International Civil Aviation Organization (ICAO) has published many rules and criteria to regulate the pronunciation for homophone words [17]. For example, ‘a’ is switched to ‘alpha’. The code switching makes ATC speech more like a dialect which is only applicable in ATC.
(d) Multilingual: In general, the ATC speech of international flights is in English, while the pilot of domestic flights usually uses local language to communication with ATCOs. For instance, Mandarin Chinese is widely used for ATC communication in China. More specifically, the Civil Aviation Administration of China (CAAC) published the ATC procedure and pronunciation in China on the basis of ICAO. Based on related ATC regulations, Chinese characters and English words are usually in one sentence of ATC speech, which generates special problems over universal ASR.

Actually, ASR has been applied to many air traffic works. Kopald et al. reviewed the importance of ASR on reducing human errors in air traffic operation [18]. Ferreiros et al. studied the speech interface for air traffic control and designed a system for voice guidance in terminals [19]. A semi-supervised learning approach was proposed to extract the semantic knowledge from the speech for improving the ASR performance in ATC [20]. Cordoba et al. presented an overview of ASR techniques and proposed an ASR system for cross-task and speaker adaptation in air traffic control [21]. An automatic speech semantic recognition system was explored to assist air traffic controller and Human-in-the-Loop (HITL) simulations were conducted to evaluate the performance of the studied system [22]. A challenge for air traffic speech recognition was held by Airbus, in which there are two tasks to be completed, i.e., speech recognition and call-sign detection [23]. Over 20 teams submitted their models to test the performance, which shows the importance of taking advantages of ASR for ATC research. The dialect ASR is also a hot research and several methods were proposed in [24].

Furthermore, only ASR outputs, i.e., computer readable texts, cannot be processed by current ATCSs effectively. ATC related elements are also needed to be extracted from ASR outputs for further processing, i.e., controlling instruction understanding (CIU). Generally, there are two types of data that implied in ATC speech, controlling intent (CI) and parameters (CPs), which is similar with the tasks for spoken language understanding. A support vector machines (SVM)-based method was proposed to infer intent of the call speech by Haffner [25], while conditional random fields (CRFs) [26] and maximum entropy Markov models [27]-based algorithms were proposed to label the semantics. Recently, deep learning-based models were proposed to finish the two tasks jointly, such as LSTM-based [28] and CNN-based [29] and recursive neural network-based [30] architectures. Experimental results showed that the models solving the two tasks jointly achieved the state-of-the-art performance for language understanding. 

Currently, there is no systematic study for sensing real-time controlling dynamics. Different techniques were used in existing works and applied to different application scenes. The listed specificities of ASR for the ATC speech are not well studied. The only way to sense real-time controlling dynamics is to train dedicated ASR and CIU models, which can cope with the above ATC specificity properly. Meanwhile, the development of techniques promotes us to study intelligent algorithms to solve the issues for obtaining better performance. In this paper, motivated by existing state-of-the-art works on ASR and CIU, we first propose a framework to solve the issue of real-time controlling dynamics sensing, i.e., converting the ATC speech to ATC related CI and CPs. In the proposed pipeline, an ASR model translates the ATC speech into computer-readable texts, which are following fed to a CIU model to extract the controlling intent and its parameters. A correct procedure is also proposed to revise minor mistakes of ASR results based on the flight information in current ATCSs, such as ADS-B or flight plan. The revised sections comprise the airline, call-sign, or controlling unit. The raw speech is first processed to spectrogram for extracting acoustic features. Learning from related works, we propose deep learning-based ASR and CIU models for the proposed framework. The ASR model contains three parts: acoustic model (AM), pronunciation model (PM), and language model (LM). The recurrent neural network (RNN)-based joint architecture serves as the main component of the CIU model to fulfill the two tasks: controlling-intent detection (CID) and parameter labelling (CPL) jointly. Some special tricks are proposed to deal with listed ASR specificities. The AM is designed to convert the ATC speech into phoneme-based labels to integrate the recognition of Chinese and English into one model. The two-dimensional convolutional operation (Conv2D) and average pooling layer (APL) are applied to mine spatial correlations of spectrogram and reduce the background noise respectively. Bidirectional LSTM (BLSTM) layers are designed to build temporal dependencies of speech frames to improve the overall performance of ASR. The PM aims at translating the phoneme-based label sequence into word-based one (Chinese character and English word), while LMs are trained to learn idiomatic rules of the ATC speech to enhance the applicability of ASR labels. The label from phoneme to word is defined as a ‘machine translation’ problem, we propose an encoder–decoder architecture to complete the conversion. Word embedding and shared BLSTM layer are designed for the CIU model, in which the two tasks can enhance the performance with each other based on ATC regulations.

Experimental results on real data show that our proposed framework and models achieve the goal of sensing real-time controlling dynamics with considerable high decoding performance and efficiency. All in all, our original contributions of this paper can be summarized as follows:

(a) A framework is proposed to obtain real-time controlling dynamics in air traffic system, which further supports ATC related applications. An ASR- and CIU-based pipeline is designed to extract ATC-related elements by deep learning models.
(b) A three-steps architecture, including AM, PM, and LM, is proposed to deal with the speech recognition in air traffic control. Our proposed ‘speech-phoneme labels-word labels’ pipeline unifies multilingual ASR (Mandarin Chinese and English in this work) into one model. By fixing the output of the AM to the basic phoneme vocabulary, the reusability of our proposed ASR model is improved greatly. Without considering dialects or other command word differences, we only need a new PM for expanding the word vocabulary.
(c) Conv2D and APL are applied to solve the complex background noise and high speech rate of the ATC speech. An encoder–decoder architecture is proposed in the PM to obtain the word sequence of the ATC speech.
(d) A BLSTM-based CIU joint model is proposed to perform the controlling intent detection and parameter labelling, in which the two tasks can enhance the performance with each other based on ATC regulations.
(e) Based on the flight information, a correction procedure is proposed to revise minor mistakes of given sections of ASR results and further improve the performance of the proposed framework.

The rest of this paper is organized as follows. The proposed framework is sketched in Section 2. The implementation of the ASR and CIU model are detailed in Section 3. The experimental configurations and results are reported and discussed in Section 4. Conclusions and future works are in Section 5.

## 2. The Proposed Framework

**Figure 1:**
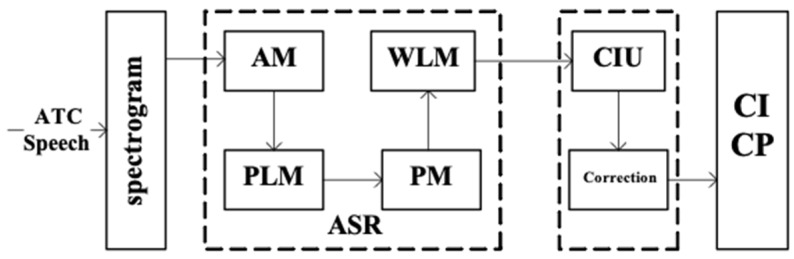
Structure of the proposed framework. ATC speech: the communication speech between air traffic controllers (ATCOs) and pilots; AM: acoustic model; PM: pronunciation model; PLM: phoneme-based language model; WLM: word-based language model; CIU: controlling instruction understanding; CI: controlling intent; CP: controlling parameter.

The proposed framework is applied to convert the ATC speech into ATC related elements, in which the ASR and CIU are core modules. Obviously, the input of the proposed framework is the ATC speech, while its output is ATC related elements, i.e., controlling intent and its parameters. The structure of the proposed framework is shown in Figure 1.

From Figure 1, we can see that the spectrogram is firstly generated from the ATC speech and serves as the input of the ASR model. There are four deep learning-based sub-models in the ASR model, AM, phoneme-based LM (PLM), PM, and word-based LM (WLM). The AM is a typical ASR model whose input and output are the spectrogram and phoneme-based label sequence respectively. Both the PLM and WLM are language models, whose difference is that they use different basic word units to model the context utterance, i.e., phoneme for PLM and word for WLM. The PLM and WLM are applied to revise the output of the AM and PM model based on characteristics of ATC application in this paper. The PM is designed as a ‘machine translation’ system, whose input and output are the phoneme-based label sequence and word-based label sequence, respectively. The CIU model takes ASR results (computer readable texts) as input to detect the controlling intent (CI) and label controlling parameters (CP). Finally, considering the flight information in current ATCSs, a correction procedure is proposed to revise minor mistakes and further improve the applicability and robustness of the sensing framework.

### 2.1. Architecture of ASR

**Figure 2:**
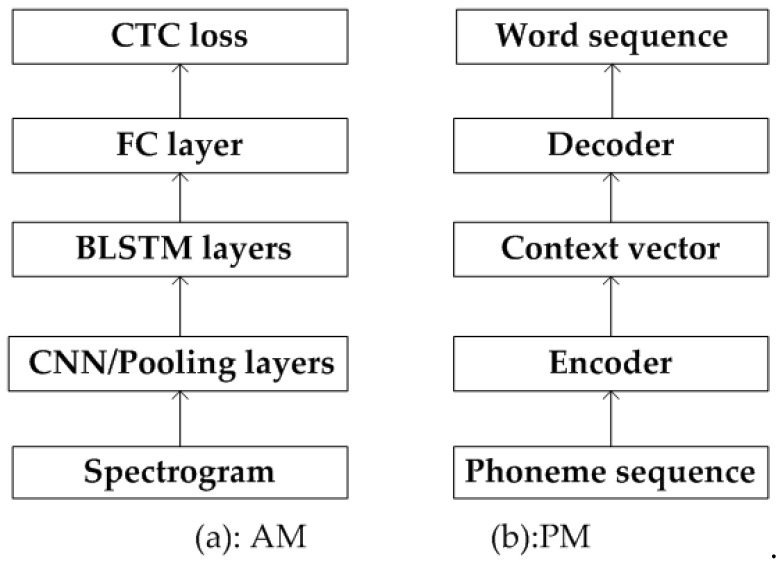
(**a**) Network of the AM; (**b**) Network of the PM. FC: fully connected; CTC: Connectionist Temporal Classification; BLSTM: bidirectional long-short term memory.

We propose a pipeline with AM, PLM, PM, and WLM sub-models in the proposed ASR model, based on which the multilingual speech recognition (Mandarin Chinese and English) can be achieved in one model. The architecture of the AM and PM are shown in Figure 2. We divide the ASR into two steps: speech to phoneme-based label sequence and further to word-based label sequence. The first one is a typical ASR problem, and we propose a CNN + BLSTM + CTC-based neural network to map the ATC speech to phoneme labels. The CNN and average pooling layer are designed to mine spatial correlations of spectrogram and filter the background noise respectively. BLSTM layers are applied to build temporal dependencies among speech frames. The CTC loss function is applied to evaluate the difference between the true labels and the predicted output, which is also known as the training of neural network [31,32]. We define the second step (phoneme labels to word labels) as a ‘machine translation’ problem since we believe that both phoneme labels and word labels are representations of the ATC speech and the only difference is using different basic units for expressing context meanings. Learning from applications of neural machine translation, an encoder and decoder network is designed to accomplish the label conversion, from phoneme to word. The encoder learns the context of the phoneme sequence in the source language (phoneme) and encodes them into a context vector, while the decoder expands the context vector in the target language (word) and decodes them into a word sequence. The LM (PLM and WLM in this work) is the standard paradigm in ASR systems and we apply the state-of-the-art RNN-based structure to model the phoneme and word rules for ATC application. From the previous four steps, the ATC speech is finally processed into computer readable texts, which is the core foundation of sensing real-time controlling dynamics.

The bottom of the AM is the input spectrogram of the raw ATC speech, which is computed by the following steps. A continuous ATC speech is firstly divided into multiple frames with 20 ms length and 15 ms shift. Since the speech rate in ATC is higher than that of speech in daily life, we use 20/15 ms for dividing speech frames compared to the 25/10 ms in universal ASR systems. Then feature engineering is used to extract features from each frame. The most classic and widely used algorithm is the MFCCs. The dimension of extracted feature depends on the configuration of filters in MFCCs. The 13-dimensional MFCCs is a typical configuration for processing speech signal. In this research, to obtain diverse features from the raw data for a more accurate translation, the basic dimension and their first- and second-order derivatives are also calculated as the input. Together with an energy dimension of the speech frame, the input spectrogram is finally determined to be 40-dimension. Moreover, the feature vectors are also normalized by subtracting the mean and dividing the variance.

### 2.2. Architecture of CIU

**Figure 3:**
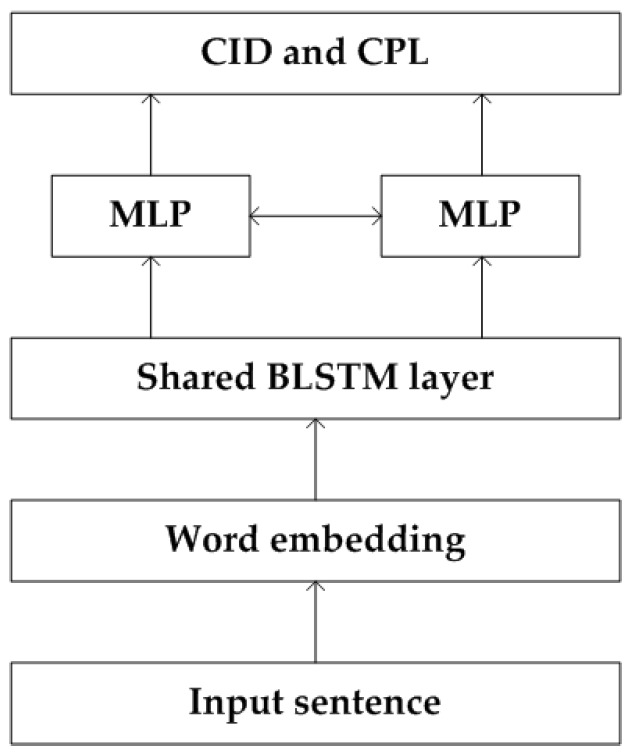
Architecture of the CIU model.

After translating the ATC speech into texts, they are only computer readable and not suitable for the processing of current ATCSs. Therefore, we propose a semantic interpretation model to bridge the ASR system and current ATCSs. By analyzing ATC related regulations and rules, we define the semantic interpretation in ATC as two tasks: controlling intent detection and controlling parameter labelling. The CID indicates concepts of the controlling instruction, while the CPL provides attributes of the controlling instruction, such as the airline and call-sign of flight, the next flight level or waypoint, et al. Technically, CID is a sequence classification task, where each sample (sentence) needs to be identified as a given intent class. CPL is a sequential labelling task, in which each word of the input sentence is classified to a class of controlling parameters. In this paper, we propose a BLSTM-based joint model to achieve the two tasks in one neural network, whose architecture is shown in Figure 3.

The input sentence of the network is the decoding result of ASR model, i.e., word sequence with Chinese characters and English words. Word embedding is a feature learning technique, in which words or phrases of the vocabulary are mapped to a word vector. The word embedding can learn abstract meanings of words and reduce the input dimension from the vocabulary size to a lower dimension. Based on ATC regulations, we design a shared BLSTM layer to extract the context utterance of the input sentence, in which the CID and CPL can enhance the performance with each other. Finally, two independent MLPs are applied to finish the two tasks. Since the output of the BLSTM layer is a sequential data, we forward the output (CI and CP) of first *i* words to the next time instant to mine temporal dependencies of the ATC speech, where i∈[1,L] and *L* is the length of the BLSTM output.

After the process of the CIU model, the controlling parameters are labelled for a specify class, such as the airline, call-sign of flight, name of controlling unit, et al. We can see that the CPs are structured data and can be received and processed by current ATCSs directly and effectively. Based on the flight information, we also propose a correction procedure to revise minor mistakes in the ASR results. For example, if the call-sign of a flight in the ASR result is 925 and there is only a flight with call-sign 9225 based on the flight plan, we can revise the 925 to 9225 to improve the reliability of our proposed sensing framework.

## 3. Methods

In this section, we introduce the techniques related to deep learning-based models in the proposed framework. The AM + PLM + PM + WLM architecture is first proposed to solve the multilingual ASR in ATC and improve the reusability for expanding the vocabulary. The Conv2D with average pooling is designed to deal with the complex noise of ATC speech under the CNN + BLSTM + CTC framework. A machine translation-based PM is also proposed to predict the word-based sequence from phoneme-based sequence. LMs are proposed to improve the decoding performance of the ASR model. In succession, the CIU model is proposed to understand the ASR output by a BLSTM + MLP-based architecture. The ATC related information is also introduced in this section.

### 3.1. Multilingual ATC

Currently, there are so many end-to-end models that are proposed to solve the universal ASR problem. However, those models mainly focus on the ASR for one language since the vocabulary of multiple languages is too large and imposes unnecessary burdens for the model training. Meanwhile, different language has different features for its word pronunciation, hence, it is difficult to model their speech words in a same ASR model directly. In this paper, we propose cascade deep learning models (AM/PM) to convert the ATC speech into computer readable texts, in which the problem of AM and PM are defined as the ASR and machine translation respectively. The AM aims at converting the ATC speech into phoneme-based label sequence, while the PM translates phoneme labels into word-based label sequence. The pipeline unifies the multilingual ASR in ATC into one framework to improve the applicability of the proposed ASR model. Basically, the phoneme label is the core pronunciation unit for all languages, which allows us to model them for AM directly when facing the multilingual ASR. The PM is likely a translation problem, i.e., mapping the context meaning of the ATC speech from a special language (phoneme) to our daily vocabulary. With our proposed model, the output of the AM is fixed at a proper set, i.e., only 300 phoneme units for the Mandarin Chinese and English speech recognition. A constant output of the AM can improve the reusability of the ASR model greatly. When we need to add new lexicon words to the ASR model, the only change is to train a new PM model without training a new AM model. In general, the architecture of the AM is deeper than the PM, which means that it needs more computational resource and time to obtain an optimized AM. By fixing the AM output, we can save computational resource and training time and reduce the influence of expanding the vocabulary of the ASR model. In the proposed ASR model, we also train a phoneme-based language model (PLM) and word-based language model (WLM) to correct decoding results of the AM and PM respectively, which further improve the overall performance of our sensing framework.

### 3.2. AM in ASR

**Figure 4:**
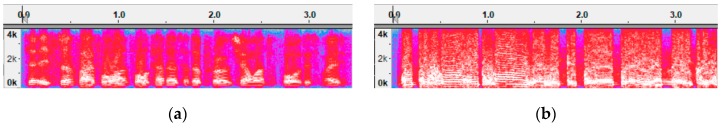
(**a**) Spectrogram of an ATC speech segment; (**b**) Spectrogram of another ATC speech segment collected from the same transmission channel with (**a**).

Based on the designed pipeline, the AM aims at converting the ATC speech into human readable texts (phoneme-based in this work). We propose a CNN + BLSTM + CTC-based architecture for the AM by learning from state-of-the-art ASR models. In order to cope with special challenges of ASR in ATC, i.e., complex background noise and high speech rate, we design the Conv2D and average pooling layers to mine spatial correlations of spectrogram and filter the background noise respectively. The Conv2D acts to extract high-level features of the input spectrogram in the time and frequency domain. A smaller filter size for time dimension is applied to adjust the high speech rate, which distinguishes the ASR in ATC from universal models (9 versus 11). Unlike the universal ASR using Max-Pooling to extract salient features of the speech, we apply the average-pooling layer to filter the background noise based on the analysis of the ATC speech. As shown in Figure 4, the two spectrograms are extracted from two speech segments from a same communication channel. In the figure, (a) is the spectrogram of an ATC speech with minor background noise, while (b) shows the spectrogram with massive noise, even covers the human speech. We can see that the intensity of the background noise is higher than that of human speech and the noise is distributed over a wide time (horizontal) and frequency (vertical) range. Therefore, the purpose of 2D average-pooling is to weaken the impacts of the background noise over human speech and further improve the performance of ASR model.

In the AM, BLSTM layers are designed to mine temporal dependencies among speech frames. Finally, the difference between the true label and network output is evaluated by the CTC loss function. CTC proposed the automatic mapping from variable length speech frames to variable length labels (phoneme in this work) by inserting ‘blank’ between any two framewise labels, which is now the standard module of end-to-end ASR models. Let the input speech $X=\langle x_{1},x_{2},\cdots ,x_{T}\rangle$ and output sequence of phoneme label $Y=\langle y_{1},y_{2},\cdots ,y_{T}\rangle$, where T is the length of speech frames. CTC predicts the probability that each speech frame belongs to a phoneme label, i.e., $y_{t}^{k}$ indicates the probability that *t^th^* frame belongs to the *k^th^* phoneme label. The output probability of all labels is normalized by the softmax function, as shown in (1). K is the label size of the AM, i.e., 300 in this work. The probability that the input speech is classified as a certain label sequence can be computed by (2) [7].

(1) $$p(k,t|x)=\exp(y_{t}^{k})/\sum_{i=1}^{K}\exp(y_{t}^{i})$$

(2) $$p(\pi |x)=\prod_{t=1}^{T}p(k,t|x)$$

By summing probabilities of all possible sequences, we can obtain the final measurement denoting the optimal label sequence for the input speech. As shown in (3), $\xi^{-1}(y)$ is the set containing all framewise labels for y. For example, both sequences, ‘abc’, ‘a_bc’, ‘a__b_c’, ‘a_b__cc_’, correspond to the final label ‘abc’, in which ‘_’ is the blank.

(3) $$p(y|x)=\sum_{\pi \in \xi^{-1}(y)}p(\pi |x)$$

### 3.3. PM and LM in ASR

As previously described, the PM is defined as a ‘machine translation’ problem, and we propose an encoder–decoder architecture to accomplish the given task. The encoder receives the input sentence as a phoneme sequence and learns context features in the source language. The decoder processes the context vector in the target language environment and outputs the word sequence. LMs are proposed to learn the semantic context of ATC application in two levels: phoneme-based (PLM) and word-based (WLM) ones. The LM predicts the probability that the next output is a certain word given the input word sequence, as shown in (4). Based on the learned context meanings, LMs are applied to correct decoding results of the AM and PM, and further improve the overall performance of the ASR model.

(4) $$p(w_{t+1}=v_{j}|w_{1}\cdots w_{t})$$

### 3.4. CIU

**Figure 5:**
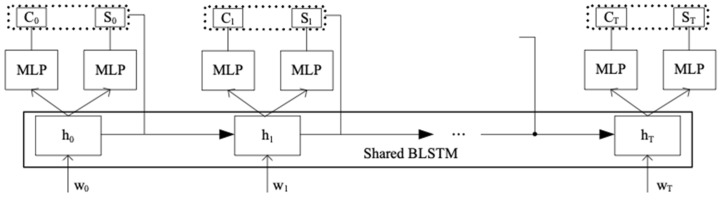
The core block of the CIU model.

Based on the ATC application, the CIU is proposed to detect the controlling intent and label its controlling parameters from the ASR output, which further supports the operational management of ATC. Let the input of the CIU is $w=\langle w_{1},w_{2},\ldots ,w_{T}\rangle$, where is the length of the ASR result, the goal of the CID and CPL can be formulated as:

(5) $$\left(\hat{c})=\arg\underset{c}{\max}\;P(c|w\right)$$

(6) $$\hat{s}=\arg\underset{s}{\max}\;P(s|w),s=\langle s_{1},s_{2},\ldots ,s_{T}\rangle$$

where c and $s_{t}$ are pre-defined ones based on the ATC application in this work. By analyzing similarities of the two tasks, we propose a BLSTM joint model to solve them in one neural network, whose core block is sketched in Figure 5. Consequently, the basic problem can be refined as (7) and (8), in which the history outputs (CI and CP) are also considered for predicting the current CI and CP labels. After labelling controlling parameters from ASR results for the ATC speech, we also propose a correction procedure based on the flight information in current ATCSs to improve the performance of the proposed sensing framework.

(7) $$p(c_{T}|w)=p(c_{T}|w_{\leq T},c_{<T},s_{<T})$$

(8) $$p(s|w)=p(s_{0}|w_{0})\prod_{t=1}^{T}p(s_{t}|w_{\leq t},c_{<t},s_{<t})$$

In this research, a joint CIU model can significantly improve the performance of the given two tasks since the ATC speech has standard communication processes. A classic ATC communication is shown in Table 1, in which there are some words for code switching, i.e., fife (five), tree (three), and tousand (thousand). From the communication we can see that the ATCO must specify the call-sign of the target flight firstly and speak details of the instruction. As to the repetition, the pilot repeats the instruction before reporting their call-sign. If the CPL successfully labels the airline and call-sign of flight at the beginning of the sentence, it will promote the CID block to confirm the role of speaker by ATC communication rules.

Finally, the correction procedure is executed by the following steps:

(a) Extracting the airline, call-sign of flight and the name of controlling unit from flight plan and ADS-B for each flight and generating a flight pool. Note that only flights in the sector of corresponding ATCOs are considered in this step.
(b) Extracting the sections to be corrected from the result of the CIU model.
(c) Comparing each section of the CIU model with corresponding information in the flight pool and selecting the most similar one as the corrected result. In addition, a similarity measurement between decoding result and corrected result is also calculated to support ATCOs to determine whether the correction can be accepted or not.

### 3.5. ATC Related

In this research, we apply the proposed framework to collect real-time controlling dynamics in a civil aviation airport in China. The ATC speech is spoken in Mandarin Chinese and English. There are 300 basic phoneme units in Mandarin Chinese and English. We also count the vocabulary appearing in the ATC speech, and there are 2414 words: 1478 Chinese characters and 936 English words. At the same time, we define the controlling intent and parameter set to support the CIU model, which contains 26 controlling intents and 55 controlling parameters respectively. Note that the defined controlling intent and parameters only cover the training samples used in this work, not a complete version for the whole ATC application. In current version, the set of the controlling intent mainly consists of the climb, cruise, descent, flying to a given point (with latitude and longitude) or waypoint, and the entry and leaving of current control area. The controlling parameter set comprises of the airline company and call-sign of the flight, target flight level, target waypoint, frequency of the radiotelephony, code of secondary surveillance radar (SSR), et al. There are two workflows in our work, model training and application. After finishing the model design, we first use historical operation data in current ATCSs, i.e., the ATC speech, to train the proposed models including the AM, PM, PLM, WLM, and CIU. Those models with optimal parameters are selected to recognition engines of the model application.

## 4. Results and Discussions

### 4.1. Data Description

In this research, we collected the raw speech from Chengdu Shuangliu international airport (ZUUU) in China. The raw speech was recorded as one file per hour continuously, and contains silence and background noise. The raw speech files are first split into segments to remove the silence and noise. Both the speech spoken by ATCOs and pilots are regarded as training samples. Currently, we use a total of 578-h ATC speech to train the proposed models in our framework, which contains 481-h Chinese speech and 97-h English speech. The duration of training samples varies from 2 to 10 s and each speech file is spoken by only one person to eliminate the vocal disturbance of different speakers. All samples are labelled manually, where each speech file has transcription labels with phonemes and words, as shown in Table 2. The bold words are Chinese characters. The word sequences are further processed to training samples of the CIU model based on the IOB label [33]. The ‘B-’ denotes the beginning of the class, while the ‘I-’ indicates that the item belongs to the same type of last ‘B-’. AL, CS are the code of airline and call-sign of the flight, and ATCN and RADAR denote the name of air traffic control unit and radar contact respectively. Meanwhile, TAXI and RW denote the taxi-way and runway in the airport respectively. HOLD indicates that the pilot should wait for the further instructions at the desired runway.

The input and output of each model in our proposed framework are summarized in Table 3, in which the phoneme and word sequence correspond to the Row 2 and 1, Table 2 respectively. The controlling intent and parameters can be obtained from Row 3, Table 2.

All 578-h ATC speech are used to train the model, in which 5% of them serve as the validation set to check the progress of the modeling training. The test data (the ATC speech) is also collected at the same airport on another two days from 8:00 a.m.–20:00 p.m. The test data is processed in the same way with that of training samples, and we finally get 8.93-h test samples to evaluate the performance of the proposed models and framework. In the test dataset, the total duration of Chinese speech and English speech are 7.10 and 1.83 h respectively.

### 4.2. Experimental Settings

The implementation codes in this work are programmed with Python, and deep learning-based models are constructed based on the Keras with TensorFlow backend. The configurations of the training server are listed as follows: 2 × Intel Core i7-6800K, 2 × NVIDIA GeForce GTX 1080 and 64 GB memory with operation system Ubuntu 16.04. Initial weights of neural networks for all models are sampled from a uniform distribution with the range [−0.1, 0.1]. The evaluation measurements in our research are listed below:

(a) WER: Word Error Rate between predicted and true labels is a common measurement of ASR applications [34], as shown below:
  (9) $$\mathrm{WER}=\frac{\mathrm{ED}}{\mathrm{len}(\mathrm{true}\_\mathrm{label})}\times 100\%$$
  where, ED means the standard edit distance, denoting minimal steps of converting one sentence into another by inserting, deleting and substitution some words. In our work, WER is applied to the AM and PM simultaneously based on their tasks.
(b) Classification precision and F1 score are applied to evaluate the performance of the CID and CPL respectively [35].
(c) RTF: Real time factor is applied to evaluate the decoding efficiency of the proposed framework. RTF is calculated as (10), in which $T_{d}$ and $D_{s}$ are the decoding time and duration of the ATC speech in seconds respectively.
  (10) $$\mathrm{RTF}=\frac{T_{d}}{D_{s}}$$

### 4.3. Test on ASR

In this section, we optimize weights and biases of the AM, PM, and LM (PLM, WLM) neural networks for the ASR model and use the test data to evaluate their performance under given measurements. Since our proposal is a cascade pipeline, we report WERs obtained by experiments with different sub-models to prove the necessity of each module, as shown in Table 4.

From the experimental results we can draw the following conclusions:

(a) AM can achieve high ASR performance from the speech to phoneme-based label sequence, i.e., 6.63% phoneme-based WER.
(b) Taking both AM and PLM results as input, additional errors are led by the PM since our proposal is a cascade pipeline, about 0.4% word-based WER.
(c) Both the PLM and WLM are proven to be useful for improving the overall ASR performance. The improvement obtained by the PLM is more significant than that of the WLM since the phoneme label is a finer-grained representation for the ATC speech compared to the word label.
(d) The proposed ASR model can translate the ATC speech into word-based label sequence with 4.04% WER. Furthermore, each sub-model is proven to be indispensable for obtaining higher ASR performance in our proposed approach.

In order to further prove the validation of our proposed ASR model, we compare WERs obtained by different ASR systems, as shown in Table 5. HMM/GMM and end-to-end methods are implemented based on the Kaldi [36] and DeepSpeech2 (DS2) [11] respectively. From experimental results we can see that deep learning-based models have superior performance over HMM/GMM model, i.e., WER 4.04% and 6.31% versus 9.14%. The performance of our proposal model is better than the word-based end-to-end model since the size of word vocabulary is too large, i.e., all 2414 words of Chinese character and English words. The WLM in our proposed model is same with the LM in DS2-based end-to-end approach to keep the fairness of the experiment. A larger output vocabulary imposes unnecessary burdens on speech modeling and usually needs more training time to be converged or even diverged. Meanwhile, RTF is applied to evaluate the decoding efficiency of different ASR models, from speech to texts. The average RTF for DS2-based end-to-end approach and our proposed ASR model are 0.139 and 0.147 respectively. Since the HMM/GMM approach is based on a different framework, we did not evaluate the RTF for it. Note that the mini-batch strategy and GPU parallel computing are applied during testing the RTF. The RTF indicates that a 10-s speech can be decoded in 1.47 s, which can meet the requirement for real-time applications.

### 4.4. Test on CIU

The main purpose of the CIU model is to detect controlling intent and label controlling parameters from ASR decoding results. With proposed evaluation measurements, the experimental results are reported in Table 6, in which we also report the results obtained by independent models for the CID and CPL to prove the validation of the proposed joint model. In addition, we also compare the obtained performance with the CNN/CRF-based model to prove the superior performance of our proposed model.

In Table 6, we can see that the proposed shared BLSTM-based CIU model can yield better performance than the CNN-based model for both CID and CPL in our application, 99.45% versus 97.93% classification precision and 97.71 versus 96.31 F1 score. We can attribute the results to the benefit of taking advantages of ATC communication rules, i.e., the input sentence (ASR decoding result) is very concise and naturally temporal dependency data. In addition, the results also show that the proposed joint model can yield higher classification precision and F1 score, which proves advantages of the joint model over independent models for CID and CPL in our work.

Finally, we replay the controlling scene (flight plan and ADS-B) of the test data to implement the proposed correction procedure. The airline, call-sign of flight and the name of controlling unit are corrected by the proposed steps. Based on experimental results, we get 0.02% WER improvement from correction compared to the raw ASR result. It is shown that our proposed ASR model has considerable high performance for converting the ATC speech into word (4.02% WER), and the proposed correction procedure can also enhance the reliability and robustness of our proposed sensing framework.

## 5. Conclusions

In this work, to obtain real-time controlling dynamics in air traffic system, we have proposed a framework to process the air traffic control speech and further support air traffic control applications. The framework consists of the ASR, CIU and correction steps and deep learning-based ASR and CIU models are proposed to achieve given goals in this framework. To cope with the specificity of ASR for the ATC speech, we propose an AM, PLM, PM and WLM pipeline to unify the multilingual ASR (Mandarin Chinese and English) into one ASR model. The AM converts the ATC speech into phoneme-based label sequence, while the PM translating them into word-based label sequence. The PLM and WLM are applied to improve the performance of the AM and PM-based on the context utterance in ATC application. In the AM, Conv2D and average-pooling layers are designed to deal with the problems of complex background noise and high speech rate. In the PM, an encoder–decoder architecture is designed to accomplish the label conversion from phoneme-based label sequence to word-based label sequence. After obtaining the word sequence from the ATC speech, we propose a CIU model to understand the controlling instruction, i.e., detect the controlling intent and label the controlling parameters. Experimental results on large amount of real data show that our proposed framework achieved given goals of sensing real-time controlling dynamics with considerable high performance and efficiency, i.e., only 4.02% WER for ASR and 0.147 RTF. Specify to the ASR model, all modules of the ASR model, AM, PLM, PM, and WLM, make contribution to improve the final ASR performance in the proposed framework. The performance of CIU is also accurate enough to complete the CID and CPL tasks, 99.45% classification precision and 97.71 F1 score. The proposed framework can be applied to other controlling scenes and languages by taking the phoneme unit as the ASR input and defining concepts and attributes for the CIU model. With the proposed framework, more real-time air traffic information can be obtained and used to support the air traffic operation by improving the safety level and operational efficiency.

In the future, we will further optimize the architecture of deep learning models in our framework to obtain better sensing performance. We will also apply sensed controlling dynamics (controlling intent and parameters) to other air traffic control applications, such as trajectory prediction, air traffic flow prediction and conflict detection etc.

## Figures and Tables

**Table 1:** Example of ATC communication.

Role	Speech Text
ATCO	China eastern fife two tree four, climb to six tousand meters
Pilot	climb to six thousand meters, China eastern fife two tree four

**Table 2:** Example of training label.

Label Type	English Sample	Chinese Sample
word	russian sky niner eight eight tree chengdu radar contact	Echo echo 八 november Charlie alpha 两 前 等 国 航 四 四 五 两
phoneme	r ah1 sh ah0 s k ay1 n ay n er ey1 t ey1 t th r iy1 ch eng2 d u1 r ey1 d aa2 r k aa1 t ae2 k t	eh1 k ow0 eh1 k ow0 b a1 n ow0 v eh1 m b er0 ch aa1 r l iy0 aw1 l f ah0 l iang3 q ian2 d eng3 g uo2 h ang2 s iy4 s iy4 uu u3 l iang3
CIU label	B-AL I-AL B-CS I-CS I-CS I-CS B-ATCN B-RADAR I-RADAR	B-TAXI I-TAXI I-TAXI B-RW I-RW I-RW I-RW B-HOLD I-HOLD B-AL I-AL B-CS I-CS I-CS I-CS

**Table 3:** Input and output of the proposed models.

Model	Input	Output
AM	spectrogram	Phoneme sequence
PLM	Phoneme sequence	Phoneme unit
PM	Phoneme sequence	Word sequence
WLM	Word sequence	Word unit
CIU	Word sequence	Controlling intent and parameters

**Table 4:** Results of different group in ASR model.

Model	AM	PLM	PM	WLM
AM/PM	6.63	-	7.01	-
AM/PM/WLM	6.63	-	7.01	6.52
AM/PLM/PM	6.63	4.12	4.50	-
AM/PLM/PM/WLM	6.63	4.12	4.50	4.04

**Table 5:** Performance of different ASR models. HMM/GMM: Hidden Markov Model and Gaussian Mixture Model; RNN: recurrent neural network; WER: word error rate; RTF: real time factor.

Methods	LM	Modeling Unit	WER (%)	RTF
HMM/GMM	3-g	Phoneme/word	9.14	-
DS2-based end-to-end	RNN-based word	Word	6.31	0.139
Our proposal	PLM and WLM	Phoneme/word	4.04	0.147

**Table 6:** Experimental results of CIU model. CNN: convolutional neural networks; CID: controlling-intent detection; CPL: controlling parameter labelling; CRF: conditional random field.

Methods	Classification Precision	F1 Score
Our proposal	99.45	97.71
Independent CID model	98.87	-
Independent CPL model	-	95.58
CNN/CRF	97.93	96.31

## Abbreviations

ADS-B	automatic dependent surveillance-broadcast
AM	acoustic model
APL	average pooling layer
ASR	automatic speech recognition
ATC	air traffic control
ATCOs	air traffic controllers
ATCSs	air traffic control systems
CI	controlling intent
CID	controlling intent detection
CIU	controlling instruction understanding
CNN	convolutional neural network
Conv2D	two-dimensional convolutional operation
CP	controlling parameters
CPL	controlling parameter labelling
CTC	Connectionist Temporal Classification
DNN	deep neural network
FC	fully connected
HMM/GMM	Hidden Markov Model/Gaussian Mixture Model
LM	language model
MFCCs	Mel Frequency Cepstral Coefficients
MLP	Multilayer Perceptron
PLM, WLM	phoneme-based LM, word-based LM
PM	pronunciation model
RNN	recurrent neural network
RTF	real time factor
